# Patient‐reported outcomes in studies of diabetes technology: What matters

**DOI:** 10.1111/dom.15858

**Published:** 2024-08-31

**Authors:** Alexandros L. Liarakos, Thomas S. J. Crabtree, Emma G. Wilmot

**Affiliations:** ^1^ Department of Diabetes and Endocrinology University Hospitals of Derby and Burton NHS Foundation Trust, Royal Derby Hospital Derby UK; ^2^ School of Medicine, Faculty of Medicine and Health Sciences University of Nottingham Nottingham UK

**Keywords:** continuous glucose monitoring, continuous subcutaneous insulin infusion, insulin pump therapy, patient reported outcomes, type 1 diabetes, type 2 diabetes

## Abstract

In recent years, diabetes technologies have revolutionized the care of people with type 1 diabetes (T1D). Emerging evidence suggests that people with type 2 diabetes (T2D) can experience similar benefits from these advances in technology. While glycaemic outcomes are often a primary focus, the lived experience of the person with diabetes is equally important. In this review, we describe the impact of diabetes technologies on patient‐reported outcome measures (PROMs). We highlight that most of the published studies investigated PROMs as secondary outcomes. Continuous glucose monitoring systems may have an important role in improving PROMs in individuals with T1D, which may be driven by the prevention or proactive management of hypoglycaemia. In people with T2D, continuous glucose monitoring may also have an important role in improving PROMs, particularly in those treated with insulin therapy. The impact of insulin pumps on PROMs seems positive in T1D, while there is limited evidence in T2D. Studies of hybrid closed‐loop therapies suggest increased treatment satisfaction, improved quality of life and decreased diabetes‐related distress in T1D, but it is unclear whether these benefits are because of a ‘class‐effect’ or individual systems. We conclude that PROMs deserve a more central role in trials and clinical practice, and we discuss directions for future research.

## Abstract 2

Please visit https://health.learning.wiley.com/courses/dom-15858/ to complete an accredited learning activity for this article and receive 1.0 *AMA PRA Category 1 Credit*™.

## 1 INTRODUCTION

Diabetes mellitus is a chronic condition, largely self‐managed, influencing quality of life (QoL) and physical health because of the burden of disease and the frequent decision‐making needed to optimize glycaemia.^1^, ^2^ The Diabetes Control and Complications Trial and the UK Prospective Diabetes Study have shown the impact of improved glycaemic control on long‐term outcomes and subsequent mortality in people with type 1 diabetes (T1D) and type 2 diabetes (T2D), respectively.^3^, ^4^, ^5^, ^6^ As a result, most diabetes studies focus on the impact of interventions on glycated haemoglobin (HbA1c) and, more recently, continuous glucose monitoring (CGM) percentage time in the glucose range.^7^

The burden of living with diabetes is substantial. Many people living with diabetes report feeling overwhelmed by the demands of diabetes and/or feel like they are failing with their diabetes management.^8^ Determining how individuals perceive diabetes impacts them by collecting data related to their symptoms, experiences, feelings and daily functioning provides knowledge around different aspects of diabetes management that glycaemic outcomes are not able to cover. Patient‐reported outcome measures (PROMs) are questionnaires completed by the person living with diabetes, allowing them to share their experiences and perspectives, capturing many aspects of the lived experience of the person with diabetes.^9^ For example, fear of hypoglycaemia is important.^10^ Hypoglycaemia is a common side effect of insulin therapy, which can cause disabling symptoms leading to a fear of recurrence.^11^ It is also important to consider the impact on how the person feels about their diabetes management. Measures of diabetes‐related distress do this by exploring how overwhelmed people feel about managing their diabetes and consider whether they feel like they are failing in their diabetes management.^10^ Beyond this, there are also measures of anxiety and depression, commonly captured using tools such as the Problem Areas in Diabetes (PAID) questionnaire.^12^ Finally, there are questionnaires that explore how satisfied people are with their glucose monitoring and treatment.^13^ Understanding these outcomes is important. If an intervention improves glycaemic outcomes but has a negative impact on PROMs, then it is important to understand this to inform person‐centred management.^14^ Specifically in diabetes, evidence suggests that routine assessment of PROMs can improve psychosocial well‐being in this population.^15^, ^16^

Technologies, such as CGM, continuous subcutaneous insulin infusion (CSII) and automated insulin delivery systems, also known as closed‐loop systems, improve HbA1c in people with T1D.^17^, ^18^, ^19^ These advances have revolutionized the management of T1D. Current glucose monitoring technology encompasses intermittently scanned CGM (isCGM), which involves sensors that need scanned to provide glucose values and real‐time CGM (rtCGM), which relates to sensors displaying glucose data on a reader or mobile phone app automatically, without the need for scanning. CSII therapies, also known as insulin pumps, deliver rapid‐acting insulin throughout the day to help manage glucose levels, while insulin boluses via the pump need to be administered for carbohydrate intake. Closed‐loop systems consist of an rtCGM device, an insulin pump and an algorithm that computes and regulates insulin delivery via the pump based on CGM‐captured glucose levels. Closed‐loop systems include ‘hybrid’ closed‐loop (HCL) therapies, which require announcements of carbohydrates and pump‐delivered meal boluses by the user, and fully closed‐loop systems, which eliminate the need for manual mealtime boluses. The available technologies have advanced at pace. For example, when isCGM launched in the UK in 2014, the sensor did not have glucose alerts for high and low levels. Fast forward to 2024 and in some countries, isCGM has now been replaced by rtCGM (without the need to scan the sensor) and has alerts. The same is true of insulin delivery. CSII therapy is becoming a thing of the past as it is superseded by HCL systems with automated insulin delivery.^20^ These rapid and substantial improvements in technology mean that we cannot assume that the findings in PROMs from even 5 years ago are relevant to the technologies available and used today.

In many countries, diabetes technologies have become the standard of care in T1D. It is becoming increasingly evident that individuals with T2D can also benefit from these advances.^21^, ^22^, ^23^ Most of the studies on diabetes technologies focus on glycaemic markers as a primary outcome, with PROMs being studied as secondary outcomes, if included at all.^24^ HbA1c as the primary outcome is often driven by the need to show the cost‐effectiveness of a given intervention. However, capturing the impact of technologies on PROMs has been suggested to be equally important to ensure empowerment and recognition of the involvement of people with diabetes in their daily management.^9^, ^24^

The 2022 narrative review from Speight et al.^24^ has been informative on the effect of glycaemic technologies on QoL and related outcomes in adults with T1D. Diabetes technologies are rapidly advancing, with more recent studies providing insight into the impact of technology on patient‐reported outcomes. The aim of our narrative review is to provide an overview of the impact of device technologies on a variety of patient‐reported outcomes in people with both T1D and T2D, including the most recently published articles in the literature.

## 2 SEARCH STRATEGY

We used the keywords ‘type 1 diabetes’, ‘type 2 diabetes’ and terms synonymous with PROMs and technology, alone and in combination, to retrieve available literature data from PubMed from inception until April 2024. Keywords for PROMs included ‘patient‐reported outcomes’, ‘person‐reported outcomes’, ‘Diabetes Distress Scale (DDS)’, ‘Diabetes Treatment Satisfaction Questionnaire (DTSQ)’, ‘Hospital Anxiety and Depression Scale (HADS)’, ‘Problem Areas in Diabetes (PAID)’, ‘EuroQoL‐5 Dimensions (EQ‐5D)’, ‘Hypoglycaemia Fear Survey (HFS)’, ‘Short Form 36 items (SF‐36)’, ‘Patient Health Questionnaire (PHQ)’ and ‘quality of life (QoL)’, Keywords for technology included ‘diabetes technology’, ‘continuous glucose monitoring’, ‘flash glucose monitoring’, ‘intermittently‐scanned continuous glucose monitoring’, ‘real‐time continuous glucose monitoring’, ‘continuous subcutaneous insulin infusion’, ‘insulin pump’, ‘closed‐loop’, ‘automated insulin delivery’ and ‘artificial pancreas’.

We included randomized trials, observational studies, systematic reviews and meta‐analyses. Key randomized trials of CGM, CSII and HCL were included based on the size of the study population and the number of PROMs reported. Our search strategy may have missed studies in which PROMs were not obvious in the title and/or abstract. Here we present the findings of some key historical publications, with a focus on the more recent additions to the literature.

## 3 CONTINUOUS GLUCOSE MONITORING

### 3.1 Type 1 diabetes

Several trials assessed the impact of CGM (isCGM or rtCGM) on PROMs in people living with T1D (PwT1D). Most of these trials investigated PROMs as secondary outcomes and are therefore underpowered to detect a significant difference in outcomes. The findings are summarized in Table 1.

**TABLE 1:** Patient‐reported outcomes from recent randomized trials of CGM in adults with diabetes.

Study (first author, year, name of trial)	Type of diabetes and study design	Intervention duration (weeks)	Study duration (weeks)	Inclusion criteria	Number of participants intervention/control	Mean age (years) intervention/control	Intervention (type of CGM) versus comparator	PROMs assessed	Results
isCGM									
Kim et al. 2024^48^	T2D 3‐arm RCT	24	24	Intensive insulin therapy HbA1c 58–108 mmol/mol (7.5%–12.0%)	52/49/47	60/57/57	Intervention: isCGM and structured education; control group 1: isCGM and conventional education; control group 2: SMBG and conventional education	DTSQ (secondary outcome)	Treatment satisfaction significantly increased from baseline in both the intervention group (MD +6.92 at week 12, MD +7.09 at week 12) and control group 1 (MD +5.29 at week 12, MD +5.97 at week 24) (*p* < 0.0001 for all) Intervention group showed a greater improvement in DTSQ compared with control group 2
IMMEDIATE; Aronson et al. 2023^22^	T2D 2‐arm RCT	16	16	Non‐insulin glucose‐lowering therapies HbA1c >58 mmol/mol (7.5%)	58/58	59/58	isCGM and DSME versus DSME	GMSS, DDS, ARMS‐D and SCPI (secondary outcomes)	Glucose monitoring satisfaction was higher in the intervention group compared with the control group (MD +0.5; 95% CI: +0.3, +0.7; *p* < 0.01) There were no treatment group differences in DDS (adjusted MD −0.2 [95% CI: −0.5, 0.2]), ARMS‐D (adjusted MD +0.4 [95% CI: −0.8, 1.6]) or SCPI scores (adjusted MD −0.2 [95% CI: −0.3, 0.2])
LIBERATES; Ajjan et al. 2023^46^	T2D 2‐arm RCT	12	12	T2D with MI on insulin or gliclazide ± other non‐insulin glucose‐lowering therapies	69/72	62/63	isCGM versus SMBG	DTSQ, EQ5D‐5L, ADDQoL (secondary outcomes)	The mean difference between isCGM and SMBG in baseline‐adjusted day‐91 DTSQ score was +1.1 point (95% CI: −0.7, +2.9) There was no difference between isCGM and SMBG in baseline‐adjusted day‐91 EQ5D‐5L utility score (mean difference −0.004; 95% CI: −0.076, +0.068) There was no overall difference in the ADDQoL score between isCGM and SMBG at day 91 (mean difference −0.02; 95% CI: −0.24, +0.20); a 1‐point difference in working life domain was observed in favour of isCGM
ISCHIA; Murata et al. 2023^26^	T1D 2‐arm crossover trial	24	32	MDI HbA1c <69 mmol/mol (8.5%)	47/46	51	Group A: isCGM + structured education, followed by SMBG versus group B: SMBG, followed by isCGM + structured education	PAID, HFS‐II (secondary outcomes)	No significant difference was observed in the change of the PAID score and HFS score between the two groups
PDF; Choe et al. 2022^23^	T2D 2‐arm RCT	12	12	Basal insulin and oral glucose‐lowering therapies HbA1c 53–86 mmol/mol (7.0%–10.0%)	63/63	59/58	isCGM plus structured education versus conventional diabetes care	SDSCA‐K (secondary outcome)	Diabetes Self‐Care, assessed with SDSCA‐K, increased in both groups with a greater extent in the intervention group (MD +4.8; 95% CI: +1.7, +8.0; *p* = 0.003)
FLASH‐UK; Leelarathna et al. 2022^27^	T1D 2‐arm RCT	24	24	MDI or CSII HbA1c 58–97 mmol/mol (7.5%–11.0%)	72/69	44/44	isCGM versus SMBG	DDS, D‐FISQ FSI, DEPS‐R, DTSQ, PHQ‐9, GMSS (secondary outcomes)	isCGM versus SMBG was associated with a higher DTSQ total score (adjusted difference, +7.0 points; 95% CI: +5.2, +8.7), higher GMSS total score (adjusted difference, +0.7 points; 95% CI: +0.5, +0.9) There were no significant changes in DDS, D‐FISQ FSI, DEPS‐R and PHQ‐9 scores between the groups
Secher et al. 2021^28^	T1D 4‐arm RCT	26	26	MDI HbA1c >53 mmol/mol (7.0%)	35/25/46/35	47	Group A: usual care Group B: automated bolus calculation Group C: isCGM Group D: isCGM + automated bolus calculation	PAID, DTSQ, DES, ADDQoL (secondary outcomes)	Compared with usual care, using isCGM + automated bolus calculation improved treatment satisfaction, psychosocial self‐efficacy and present life quality Treatment satisfaction improved by using isCGM alone versus usual care
rtCGM									
Steno2tech; Lind et al. 2024^50^	T2D 2‐arm RCT	52	52	Insulin therapy (≥1 insulin injection/day) HbA1c ≥58 mmol/mol (7.5%)	40/36	ND	rtCGM versus SMBG	WHO‐5, DDS, Hypoglycaemia Fear Survey, DTSQ, GMSS, Swedish National Board of Health and Welfare questionnaire for Physical Activity, Danish Perceived Dietary Adherence questionnaire (secondary outcomes)	CGM versus SMBG was associated with greater self‐rated diabetes‐related health, well‐being, satisfaction and health behaviour
PACE; Rilstone et al. 2024^38^	T1D 2‐arm crossover trial	6 (40 days)	12 (90 days)	MDI or CSII + regular exercise	17	35	Group A: rtCGM with urgent lower soon alert on followed by alert off Group B: rtCGM with urgent lower soon alert off followed by alert on	DDS‐17, HFS‐II (secondary outcomes)	There was no significant difference in the change of quality of life from baseline by using the ‘alert on’ modality compared with the ‘alert off’ modality
Uduku et al. 2023^33^	T1D 2‐arm RCT	12	12	MDI + history of SH requiring emergency medical services	15/8	40/48	rtCGM versus SMBG	DTSQ, PAID, HFS‐II (secondary outcomes)	Participants randomized to receive rtCGM reported significantly improved within‐group median (IQR) DTSQ scores [29.5 (28–33.3) at baseline versus 38 [37–39] at 12 weeks, *p* < 0.01], but no significant difference was observed between groups No significant changes were seen within or between groups for diabetes‐specific distress (PAID), and fear of hypoglycaemia (HFS‐II)
Yoo et al. 2022^37^	T1D 2‐arm RCT	Intervention group: 12 weeks control group: 24 weeks	24	MDI HbA1c 53–97 mmol/mol (7.0%–11.0%)	23/22	38/39	Intervention group: rtCGM with education; control group: rtCGM with no education (followed by a 12‐week extension phase with education)	DTSQ (secondary outcome)	Intervention group versus control group was associated with higher DTSQ satisfaction score (mean difference +4.3; +1.4, +7.2; *p* = 0.005) and lower DTSQ hypoglycaemia score (mean difference −1.2; −1.9, −0.4; *p* = 0.003) at week 12 DTSQ satisfaction remained similar between baseline and week 12 in the control group, while DTSQ score increased significantly by +3.0 points (95% CI: +1.0, +5.0; *p* = 0.009) after 3 months of education of the control group There was no significant difference in DTSQ hyperglycaemia score between the two groups at week 12 However, the mean score improved from 3.7 ± 2.5 to 2.6 ± 1.6 with a difference of −1.0 (95% CI: −2.0, −0.5; *p* = 0.015) in the educated control group from week 12 to week 24
Moon et al. 2023^53^	T2D 3‐arm RCT	1–2 (group 1: 1 week at baseline, group 2: 1 week at baseline and 1 week at week 12)	24	Non‐insulin glucose‐lowering therapies HbA1c 58–86 mmol/mol (7.5%–10.0%)	18/15/15	56/54/51	Group 1: one session of rtCGM; group 2: two sessions of rtCGM with a 3‐month interval between sessions Control group: SMBG	K‐DMSES, ADS‐K, SDSCA‐K (secondary outcomes)	For K‐DMSES, the total score did not show statistical significance in groups 1 and 2 versus SMBG; however, the nutrition subscale significantly improved in group 2 SMBG (adjusted difference +6.63; 95% CI: +1.27, +12.0; *p* = 0.018) There were no significant differences in ADS‐K and SDSCA‐K scores in groups 1 and 2 versus SMBG
MOBILE Martens et al. 2021^51^	T2D 2‐arm RCT	32	32	Insulin (1 or 2 daily injections of long‐ or intermediate‐acting basal insulin without prandial insulin) ± non‐insulin glucose‐lowering therapies HbA1c 62–102 mmol/mol (7.8%–11.5%)	116/59	56/59	rtCGM versus SMBG	CGMSS (secondary outcome)	High treatment satisfaction In the CGM group, the mean score on the CGM satisfaction scale was 4.1, with mean scores of 4.2 on the benefits subscale and 1.9 on the hassles subscale
rtCGM versus isCGM									
Gupta et al. 2024^43^	T1D 3‐arm crossover trial	4	24	MDI HbA1c 64–108 mmol/mol (8.0%–12.0%)	17/17/34	23/21/29	Group A: rtCGM for 2 weeks followed by isCGM for 2 weeks at 3 months; group B: isCGM for 2 weeks followed by rtCGM for 2 weeks at 3 months; group C: SMBG	QOLID (secondary outcome)	Quality of life improved in groups A and C at 3 months compared with baseline There was no significant difference in quality of life among the 3 groups at 3 months
ALERTT1 Visser et al. 2021, 2023^40^, ^41^	T1D 2‐arm RCT (with extension phase)	108	108	Insulin therapy + previous isCGM use	117/112	ND	Intervention: rtCGM with alerts versus control: isCGM without alerts (switching to rtCGM at 6 months)	HFS‐II (secondary outcome)	At 6 months, rtCGM with alerts versus isCGM without alerts was associated with improved HFS‐II worry score (15.4 versus 18.0, *p* = 0·0071) In the extension phase, the change in HFS‐worry score was −5.17 points (month 24 versus month 0; *p* < 0.0001) in the intervention group and −2.67 (month 24 versus month 6; *p* = 0.0008) in the control group

Abbreviations: ADDQoL, diabetes quality of life; ADS‐K, Appraisal of Diabetes Scale–Korean; ARMS‐D, Adherence to Refills and Medications–Diabetes; CGM, continuous glucose monitoring; CGMSS, Continuous Glucose Monitoring Satisfaction Scale; CI, Confidence Interval; CSII, continuous subcutaneous insulin infusion; DDS, Diabetes Distress Scale; DEPS‐R, Diabetes Eating Problem Survey–Revised; DES, Diabetes Empowerment Scale; D‐FISQ FSI, Diabetes Fear of Injecting and Self‐Testing Questionnaire, Fear of Self‐Injection component; DTSQ, Diabetes Treatment Satisfaction Questionnaire; EQ5D‐5L, Euro Quality of Life 5 Dimension; GMSS, Glucose Monitoring Satisfaction Survey; HFS‐II, Hypoglycaemia Fear Survey‐II; isCGM, intermittently scanned continuous glucose monitoring; K‐DMSES, Korean Diabetes Management Self‐Efficacy Scale; MD, mean difference; MDI, multiple daily insulin injections; ND, no data available during literature review; PAID, Problem Areas In Diabetes; PHQ‐9, Patient Health Questionnaire 9‐item version; PROMs, Patient‐Reported‐Outcome Measures; QOLID, Quality of Life Instrument for Indian Diabetes Patients; RCT, randomized controlled trial; rtCGM, real‐time continuous glucose monitoring; SCPI, Skills, Confidence and Preparedness Index; SDSCA‐K, Summary of Diabetes Self‐Care Activities questionnaire‐Korean version; SH, severe hypoglycaemia; SMBG, self‐monitoring of blood glucose; T1D, type 1 diabetes; T2D, type 2 diabetes; WHO, World Health Organization.

#### 3.1.1 Intermittently scanned continuous glucose monitoring

The IMPACT trial^25^ was the first randomized controlled trial (RCT) of isCGM in people with T1D. It showed that in those with baseline HbA1c levels of ≤58 mmol/mol (7.5%), isCGM compared with self‐monitoring of blood glucose (SMBG) improved hypoglycaemia (primary outcome) and was associated with an improvement in treatment satisfaction and perceived frequency of hyperglycaemia. There were no between‐group differences for fear of hypoglycaemia, QoL or diabetes‐related distress. In 2023, the ISCHIA Study Group^26^ published their two‐arm crossover randomized trial, which compared isCGM and structured education with SMBG. While hypoglycaemia improved, the authors found no significant difference in diabetes‐related distress or fear of hypoglycaemia between the two groups.

The 2022 FLASH‐UK RCT^27^ compared isCGM with SMBG in PwT1D treated with CSII or multiple daily injections of insulin (MDI) and showed that isCGM with optimal alarms improved HbA1c (primary outcome) and was associated with a higher treatment and glucose monitoring satisfaction. No significant changes in diabetes‐related distress, depression, or fear of self‐injecting between the groups were observed. After 26 weeks, the four‐arm RCT^28^ in PwT1D with baseline HbA1c >53 mmol/mol (7.0%) showed that, compared with usual care, using isCGM with automated bolus calculation improved treatment satisfaction, psychosocial self‐efficacy and QoL. Treatment satisfaction also improved by using isCGM alone compared with usual care.

#### 3.1.2 Real‐time continuous glucose monitoring

The DIAMOND trial^29^ was a 24‐week RCT assessing the impact of rtCGM compared with usual care, with HbA1c as the primary outcome. The authors showed that rtCGM versus SMBG improved HbA1c and was associated with reduced diabetes‐related distress and increased confidence in managing hypoglycaemia. There were no between‐group differences in general emotional well‐being, general health status or fear of hypoglycaemia observed in the trial. The RCT of Pratley et al.^30^ showed that rtCGM versus SMBG was not associated with changes in fear of hypoglycaemia, diabetes distress, hypoglycaemia awareness or emotional well‐being. However, the GOLD crossover trial^31^ showed an increase in treatment satisfaction, emotional well‐being and confidence in managing hypoglycaemia in PwT1D using rtCGM compared with SMBG.

The CONCEPTT RCT^32^ showed the clear glycaemic benefits of rtCGM compared with SMBG in women aged 18–40 years with T1D who were pregnant or planning pregnancy. Although there were no between‐group differences in any PROMs at the end of the trial, there were group × time interactions favouring CGM for fear of hypoglycaemia and glucose monitoring satisfaction both during pregnancy and pregnancy planning.

PwT1D are often excluded from RCTs if they have a history of severe hypoglycaemia (SH). However, those with problematic hypoglycaemia are arguably the group with the most to gain from access to rtCGM with alarms to alert them to hypoglycaemia. Some studies evaluated the role of rtCGM in this important subgroup. The RCT of Uduku et al.^33^ showed that among PwT1D, with a history of SH requiring emergency medical services, rtCGM users reported significantly improved treatment satisfaction at week 12 compared with baseline, but no significant difference was observed between the rtCGM and SMBG groups. In the IN CONTROL crossover trial,^34^ which involved PwT1D, treated with MDI or CSII, and a history of impaired awareness of hypoglycaemia (IAH), fear of hypoglycaemia was lower in the rtCGM group compared with the SMBG group. However, no differences were observed in IAH, diabetes distress, diabetes self‐care, general emotional well‐being, or health status between the two groups. The HypoDE RCT^35^ assessed the impact of rtCGM in adults with a history of IAH or SH. Hypoglycaemia improved, and although there was a trend towards improvements in PROMs with rtCGM compared with SMBG at 6 months, between‐group differences were observed only for diabetes‐related distress and satisfaction with rtCGM. Similar results were shown in the HypoCOMPaSS RCT,^36^ which assessed the impact of rtCGM versus SMBG in PwT1D with IAH and recurrent SH episodes. The authors showed that treatment satisfaction improved, and fear of hypoglycaemia decreased across the whole cohort at 6 months and was maintained at 24 months. However, there were no between‐group differences in these PROMs, suggesting the importance of education.

The 2022 RCT by Yoo et al.^37^ assessed the effect of education in rtCGM users with T1D and baseline HbA1c 53–97 mmol/mol (7.0%–11.0%). The authors showed that rtCGM combined with education (intervention group) was associated with significantly lower hypoglycaemia scores and higher diabetes treatment satisfaction at week 12 compared with the control group (no education). The 2024 PACE study^38^ was a crossover trial in PwT1D assessing the impact of rtCGM with a predictive hypoglycaemia alert function on the frequency, duration and severity of hypoglycaemia occurring during and after regular (≥150 min/week) physical activity. Contrary to what one might imagine, the study showed that the predictive hypoglycaemia alert function was not associated with changes in fear of hypoglycaemia or diabetes distress; however, the low Gold score and hypoglycaemia fear at baseline, and the low number of completed questionnaires may have influenced the results observed.

A number of studies have compared the impact of isCGM with rtCGM. The RCT of Reddy et al.^39^ compared the effect of rtCGM alerts versus isCGM without alerts in PwT1D with a history of IAH or SH. The authors found that compared with isCGM, rtCGM was associated with a significant reduction in fear of hypoglycaemia (HFS‐II total and worry subscale), but within‐ or between‐group differences for diabetes distress or HFS‐II behaviour subscale were not observed. The ALERTT1 RCT^40^ also compared CGM with alerts with isCGM without alerts in PwT1D. It showed that rtCGM use was associated with a significant improvement in time in range (primary outcome) and fear of hypoglycaemia (secondary outcome) at 6 months compared with isCGM. Upon completion of the 6‐month period, the control group switched to rtCGM, and the intervention group continued rtCGM up to 24 months (extension phase). The authors showed that there were significant improvements in the fear of hypoglycaemia in the intervention group and control group during the extension phase.^41^ The CORRIDA trial also found rtCGM to be superior to isCGM for improving the time in range; however, no significant changes in QoL were observed.^42^ A more recent RCT of short‐term use of CGM from Gupta et al.^43^ did not detect any difference in QoL when comparing rtCGM with isCGM.

Overall, CGM can have an important role in improving PROMs in PwT1D, which may be driven by the prevention or proactive management of hypoglycaemia. However, this needs further assessment in trials involving PROMs as primary outcomes in the future.

### 3.2 Type 2 diabetes

Similar to T1D, most clinical trials describing the impact of CGM on PROMs in people living with T2D (PwT2D) assess these measures as secondary outcomes, which makes the findings exploratory rather than confirmative. Table 1 summarizes the findings of recent RCTs.

#### 3.2.1 Intermittently scanned continuous glucose monitoring

The first RCT of isCGM in people with MDI‐treated T2D was conducted by Haak et al.^44^ There was no between‐group difference in HbA1c (primary outcome). However, treatment satisfaction (secondary outcome), assessed using the diabetes treatment satisfaction questionnaire, was higher in the isCGM group compared with the SMBG group. One of the few trials to assess treatment satisfaction as the primary outcome was the RCT from Yaron et al.,^45^ which compared isCGM with SMBG in an MDI‐treated T2D population with baseline HbA1c 58–86 mmol/mol (7.5%–10.0%). Secondary outcomes included the change in diabetes‐dependent QoL. The authors found that isCGM versus SMBG did not lead to a significant improvement in treatment satisfaction. However, isCGM users found the device to be more flexible and would recommend it to their counterparts compared with SMBG. Most other trials included PROMs as a secondary outcome. The LIBERATES trial^46^ published in 2023 recruited PwT2D with a recent myocardial infarction who were treated with insulin or gliclazide. There were no significant differences in treatment satisfaction or QoL between isCGM and SMBG.

The effect of isCGM in non‐insulin‐treated T2D was assessed in the 2020 RCT from Wada et al.^47^ The authors found that isCGM was associated with a significant improvement in treatment satisfaction compared with SMBG. The more recent IMMEDIATE trial^22^ was another RCT in non‐insulin‐treated PwT2D, which compared isCGM and diabetes self‐management education (intervention) with diabetes self‐management education (control). At week 16, glucose monitoring satisfaction was significantly higher in the intervention group compared with the control group, but there were no between‐group differences in diabetes distress, diabetes medication adherence or confidence and preparedness for diabetes self‐management.

The PDF RCT^23^ assessed the effect of isCGM and structured education in PwT2D treated with basal insulin and/or oral glucose‐lowering therapies. A simple visual tool was utilized to assess the impact of food on postprandial glucose (intervention) compared with conventional diabetes care (control). The authors showed that diabetes self‐care increased in both groups but significantly more in the intervention group. The recent 24‐week RCT by Kim et al.^48^ also highlighted the importance of combining isCGM with structured education in the T2D population treated with intensive insulin therapy. They showed that isCGM, along with structured education, was associated with a greater improvement in treatment satisfaction compared with SMBG and conventional education.

#### 3.2.2 Real‐time continuous glucose monitoring

The advances in diabetes technologies over the past decade have been substantial. An earlier RCT by Tang et al.^49^ included PwT2D treated with insulin alone or in combination with oral glucose‐lowering therapy. They assessed the impact of rtCGM on treatment satisfaction (primary outcome). At 24 weeks, rtCGM use was associated with significantly lower overall treatment satisfaction compared with SMBG. However, this RCT was conducted a decade ago. The available technologies have advanced greatly during the past decade in terms of sensor size, duration of sensor life, calibration requirements, reliability and accuracy. Hence, it is probable that the older technology used in the trial may have influenced the outcomes reported.

In contrast, a recently published 12‐month RCT from the Steno Diabetes Centre,^50^ which included PwT2D treated with insulin therapy (≥1 insulin injection/day), showed that rtCGM improved glucose time in range and was associated with greater self‐rated diabetes‐related health, well‐being, satisfaction and health behaviour compared with SMBG. Similarly, the MOBILE RCT,^51^ which showed that rtCGM improved HbA1c, also showed high treatment satisfaction with rtCGM use in a basal insulin‐treated T2D population over 32 weeks. In the DIAMOND RCT,^52^ although rtCGM versus SMBG improved HbA1c, rtCGM use was not associated with significant changes in hypoglycaemia awareness, diabetes distress, fear of hypoglycaemia or QoL in PwT2D treated with MDI. However, high satisfaction with CGM use was reported.

There are a limited number of RCTs in the non‐insulin‐treated T2D population assessing the effect of rtCGM on PROMs. In the trial of Moon et al.,^53^ the short‐term use (1 week or 2 weeks) of rtCGM was not associated with significant changes in diabetes management self‐efficacy, diabetes self‐care or appraisal of diabetes compared with SMBG. However, in the RCT of Cox et al.,^54^ the 8‐week use of rtCGM versus SMBG was associated with significantly improved QoL, glucose monitoring satisfaction and diabetes distress.

In summary, CGM may play an important role in improving PROMs in PwT2D, particularly in those treated with insulin therapy. Further research to investigate the impact of CGM on PROMs in the non‐insulin‐treated T2D population as well as trials assessing PROMs as a primary outcome in T2D are needed.

## 4 CONTINUOUS SUBCUTANEOUS INSULIN INFUSION

### 4.1 Type 1 diabetes

Much of the evidence underpinning the use of CSII is historical; more recent studies are limited because of the shift in focus towards HCL therapy. Again, PROMs were usually included as secondary outcome measures.

The HypoCOMPaSS trial by Little et al.,^55^ utilized a 24‐week 2 × 2 factorial crossover RCT to assess the impact of pump therapy versus MDI in individuals randomized to either SMBG or rtCGM on hypoglycaemia awareness, fear of hypoglycaemia and SH. It also assessed diabetes treatment satisfaction. Although rates of SH were reduced and hypoglycaemia awareness was improved with the use of CSII (irrespective of glucose monitoring modality), this was similar across all treatment regimens. Diabetes treatment satisfaction was, however, highest in the CSII/rtCGM group. The REPOSE cluster RCT of CSII compared with MDI also described higher treatment satisfaction and diabetes‐specific QoL.^56^

Although initially the RCT by DeVries et al.^57^ was intended to utilize a crossover design, because of high rates of drop‐out in individuals initially assigned to CSII at the crossover phase, the study reported only outcomes from the first phase of the trial. This 16‐week RCT showed reductions in HbA1c and also assessed patient reported outcomes via the DTSQ and Medical Outcome Study 36‐item Short Form Survey (SF‐36). While no significant change in treatment satisfaction was noted between the two groups, the CSII group experienced significant improvements in the ‘general health’ sub‐scores on SF‐36 in comparison with those continuing with MDI.

A 2016 online survey of 115 CSII users noted a decrease in both the number and severity of hypoglycaemic episodes, with no increases in anxiety, worry or time off work.^58^ Notably, this survey also highlighted potential downsides of CSII, including issues with both insertion sites and technical issues, which require additional support from health care professionals. However, no validated metrics were used as part of this survey. Finally, a recent observational study assessing a tubeless pump system showed improvements in patient reported QoL and high levels of user satisfaction in both individuals new to CSII and those switching from alternative tubed systems.^59^

The available evidence supports that pump therapy is probably to have a positive impact on PROMs. Because of the increase in availability and clinical usage of HCL systems, focus on research has shifted to these systems rather than CSII, and further work in this area is therefore probably limited.

### 4.2 Type 2 diabetes

There are multiple studies reporting PROMs in people with T2D with CSII. RCTs comparing CSII to MDI in people with T2D have shown improvements in HbA1c and diabetes treatment satisfaction scores.^60^, ^61^ However, others have shown no significant difference in treatment satisfaction between MDI and CSII,^62^ although, again in all of these studies, PROMs have been a secondary rather than primary outcome, limiting the conclusions that can be drawn. The VIVID study compared MDI versus CSII administered U500 insulin and noted a significantly greater Treatment Related Impact Measure for Diabetes score in the CSII group compared with the MDI group. There were no between group differences observed in the Treatment Related Impact Measures for Diabetes Device scores.^63^

Real‐world evidence reporting PROMs in people with T2D does exist but is again limited. The 2011 pilot cohort study by Frias et al.^64^ showed improvements in health‐related QoL and treatment satisfaction. In addition, they explored associations between glycaemic outcomes and PROMs. They reported an association between decreased HbA1c and improved treatment satisfaction but no other associations. Higher time‐in‐range assessed by either rtCGM or SMBG was associated with improvements in both treatment satisfaction and health‐related QoL with CSII in individuals with T2D.^65^

There is some evidence to support the use of CSII to improve PROMs in individuals with T2D, including the impact on compliance with insulin therapy. However, access to CSII for individuals with T2D in most health care systems is limited and, given the increasing use of HCL therapy, it seems unlikely that exploring PROMs with CSII outside of HCL in individuals with T2D further will be of benefit.

## 5 CLOSED‐LOOP

### 5.1 Type 1 diabetes

There are multiple RCTs exploring a range of different HCL insulin delivery systems, most of which reported a range of PROMs as a secondary outcome.^66^, ^67^, ^68^, ^69^, ^70^, ^71^, ^72^ These are summarized in Table 2. Many of these were also included in the recent network meta‐analysis by Pease et al.,^73^ which showed possible improvements in QoL with HCL compared with MDI.

**TABLE 2:** Patient‐reported outcomes from recent randomized trials of hybrid closed‐loop therapy in adults with type 1 diabetes.

Study (first author, year, name of trial)	Study design	Intervention duration (weeks)	Study duration (weeks)	Inclusion criteria	Number of participants intervention/control	Mean age (years) intervention/control	Intervention versus comparator	PROMs assessed	Results
Kim et al. 2024^66^	2‐arm RCT	12	12	South Korea HbA1c <86 mmol/mol (10.0%)	53/51	40/39	HCL versus SAP	DTSQ, ITSQ	Improvements in DTSQ in both arms with no statistically significant difference ITSQ improved in both arms but was greater in HCL arm
Schneider‐Utaka et al. 2023^67^	2‐arm crossover RCT	16	32	Age ≥60 CSII use ≥3 months at baseline HbA1c ≤86 mmol/mol (10.0%)	37	67	HCL versus SAP	WHO‐5, DDS, GMSS, INSPIRE, HCS	Improved DDS total score, powerless subscale score and physician distress score during HCL period Improved Trust subscale score GMSS during HCL period
iDCL; Kudva et al. 2021^68^	2‐arm RCT	26	28–34	Age 14–71 HbA1c 36‐92 mmol/mol (5.4‐10.6%)	56/56	33/33	HCL versus SAP	HFS‐II, DDS, HAS; HCS; Clarke; INSPIRE; SUS; TAQ	At 26 weeks, the HCL had lower DDS scores and improvements in nine HCS domains High SUS and TAQ in both arms. No differences in other PROMs
Hood et al. 2021^69^	2‐arm crossover RCT	12	28	HbA1c 42–96 mmol/mol (7.0%–11.0%)	113	19	HCL versus 1st‐generation HCL (670G)	DDS, HCS, GMSS, TAQ	Improvements in GMSS during HCL period versus 670G

Abbreviations: CGM, continuous glucose monitoring; Clarke, Clarke Hypoglycaemia Awareness Score; CSII, continuous subcutaneous insulin infusion; DDS, Diabetes Distress Scale; DTSQ, Diabetes Treatment Satisfaction Questionnaire; GMSS, Glucose Monitoring Satisfaction Survey; HAS, Hypoglycaemia Avoidance Scale; HCS, Hypoglycaemic Confidence Scale; HCL, Hybrid Closed‐Loop; HFS‐II, Hypoglycaemia Fear Survey‐II; INSPIRE, Insulin Delivery Systems: Perceptions, Ideas, Reflections, Expectations Survey; ITSQ, Insulin Treatment Satisfaction Questionnaire; PROMs, Patient‐Reported‐Outcome Measures; RCT, randomized controlled trial; SAP, sensor augmented pump therapy; SUS, System Usability Score; TAQ, Technology Acceptance Questionnaire; WHO, World Health Organization.

Within the RCTs summarized in Table 2, notable positive effects of HCL included improvements in diabetes‐related distress, hypoglycaemic confidence and general and diabetes‐related QoL. Notably, the outcomes across trials are inconsistent, with some reporting no change in PROMs and others reporting changes in specific subscale scores only. It is also important to reflect that commercial HCL systems became available from 2017. As with many technologies, as the years advance, the systems improve, and this would probably impact the lived experience of people using the systems and the associated PROMs. There are also differences between the HCL systems and how these differences impact PROMs, which will need to be explored further in future work.

In terms of system‐specific insights, a small post‐hoc analysis of a Medtronic 780G RCT, including 41 individuals, noted significant reductions in anxiety and a shift towards becoming more emotionally aware and less self‐blaming in stressful situations.^74^

Some observational studies have been proven informative. Tubeless systems were explored in a single‐arm prospective cohort study and associated with significant improvements in diabetes‐related distress, hypoglycaemic confidence and diabetes treatment satisfaction after 3 months.^75^ Real‐world evidence from the 2021 survey of 1435 Control‐IQ users by Pinsker et al., showed high levels of user satisfaction, improved QoL, and ease of use across a range of metrics.^76^ Key factors felt by users to contribute to this included high levels of CGM accuracy, improved diabetes control, reduction in extreme blood glucose levels and improved sleep quality.

The real‐world outcomes from the NHS England closed‐loop pilot reported in 2023 showed reductions in diabetes‐related distress, the Gold Score and positive user experiences compared with previous therapy and included a mixture of all HCL systems commercially available in the United Kingdom at the time of undertaking.^19^ This makes it particularly notable for identifying a possible ‘class‐effect’ rather than the impact of a particular HCL system. The idea of a ‘class‐effect’ is also suggested by a recent multicentre prospective cohort study by Beato‐Víbora et al., which showed improvements in QoL, treatment satisfaction and sleep from baseline in individuals commencing both the Control‐IQ and the Medtronic 780G systems, but with no statistically significant difference in these between the systems.^77^

Finally, PROMs were also explored in specific subgroups of adults with T1D using HCL. The 2021 single‐arm crossover study by Bisio et al.^78^ showed both improvements in treatment satisfaction scores and QoL in older adults with T1D.

While robust conclusions cannot be drawn from observational data, there are consistencies between the randomized control trial and real‐world evidence. Overall, HCL in adults with T1D would probably improve PROMs, although further work to explore the impact of HCL across multiple systems with different features will need to continue.

### 5.2 Type 2 diabetes

At the time of writing, there are few RCTs reporting PROMs in people with T2D using closed‐loop systems. These studies report mixed results. One crossover study showed no improvements in PROMs, including anxiety and depression, as measured by PAID, and hypoglycaemia fear with a fully closed‐loop compared with standard therapy, and actually reported increases in Hypoglycaemia Worry Scores with a closed‐loop.^79^ A further crossover randomized study in individuals undergoing dialysis randomized to standard care or fully closed‐loop sequentially found higher hypoglycaemic confidence in the closed‐loop phase, but no other significant differences in PROMs.^80^ In addition, there is a paucity of robust real‐world evidence. Insulin optimization remains a barrier to achieving target HbA1c levels. Fully closed‐loop presents the opportunity to improve outcomes with reduced reliance on health care professionals for dose optimization given the mealtime insulin is automated, in addition to the basal.

Future studies evaluating the clinical utility of HCL in T2D should include PROMs as part of a holistic assessment of these technologies alongside glycaemic metrics and HbA1c. Studies to date are small and currently access to HCL for PwT2D in most health care systems is extremely limited. The clinical utility of automated insulin delivery in T2D is promising but remains underexplored. The difference these systems make to the lived experiences of PwT2D, measured using PROMs, will be crucial in developing our understanding of the acceptability and impact.

## 6 OTHER TECHNOLOGY

‘Do‐It‐Yourself’ closed‐loop systems were developed just under a decade ago by those in the diabetes community with technological knowledge and a frustration at the lack of access to closed‐loop technology. A movement referred to as #wearenotwaiting. The use of open source ‘Do‐It‐Yourself’ systems increased rapidly in the years before the wider availability of commercial closed‐loop systems. Early work by the #wearenotwaiting community identified improvements in QoL, quality of sleep and significant psychosocial benefits.^81^, ^82^ Many of these studies did not utilize validated PROMs. A more recent cross‐sectional study by Schipp et al.^83^ explored multiple psychosocial outcomes and PROMs comparing open‐source HCL versus non‐users and showed higher treatment satisfaction, improved sleep quality, lower diabetes distress and lower fear of hypoglycaemia in the open‐source HCL cohort. Schipp et al. concluded that those using open‐source closed‐loop systems had better psychosocial outcomes than those not using the systems after adjusting for sociodemographic and clinical characteristics.

Fully closed‐loop insulin delivery using dual hormone systems may also be on the horizon, but research in this area is in its infancy and beyond the scope of this review. Work in this area should consider, including PROMs, particularly in the later phases of clinical trials.

## 7 DISCUSSIONS

**FIGURE 1:**
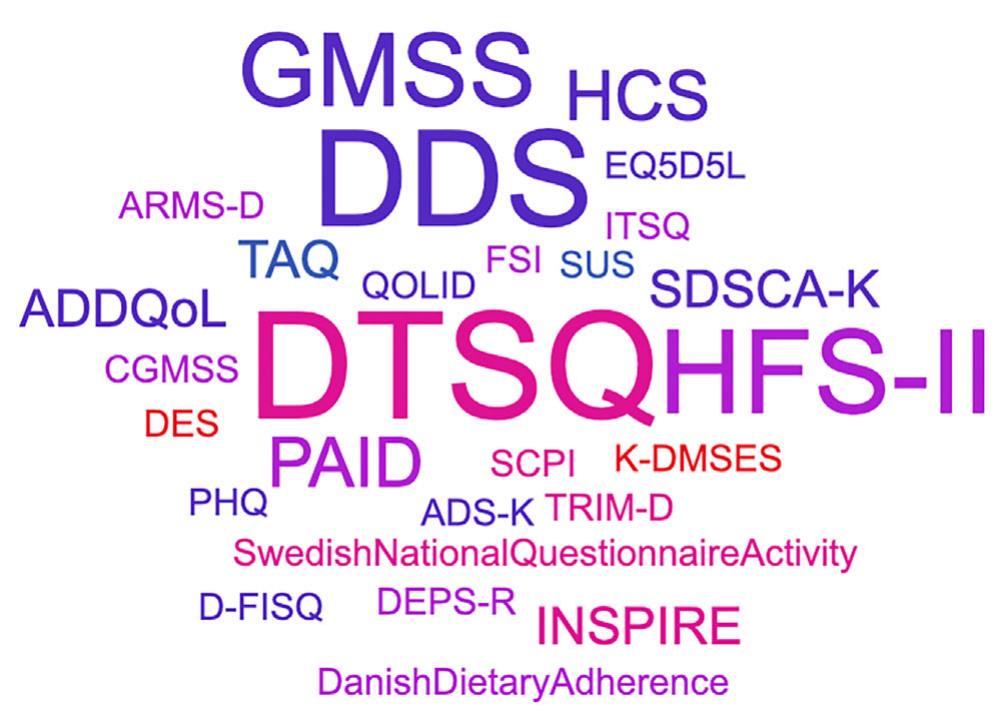
Tools assessing patient‐reported outcome measures (see Tables 1 and 2 for definitions).

Understanding the impact of diabetes technologies on the lived experience of the person with diabetes is paramount. A variety of validated PROMs are available to support us in this aim. However, heterogeneity in the tools used (Figure 1) to assess PROMs, their inclusion as secondary rather than primary outcomes combined with the rapid evolution of the available technologies presents challenges in understanding the impact across studies and any changes or advances in PROMs over time. Often studies examine PROMs as secondary outcomes, limiting our ability to make meaningful interpretation as the trials are not sufficiently powered to assess for changes in these endpoints. This highlights the need for adequately powered studies investigating PROMs as primary outcomes. To overcome the heterogeneity in the tools used consensus from both health care professionals and people living with diabetes on the most valuable tools in the context of diabetes technology would be welcomed.

With the caveat of the above, the current evidence indicates that CGM may have an important role in improving PROMs in PwT1D, while rtCGM versus isCGM is associated with a reduced fear of hypoglycaemia. These benefits of CGM systems may be driven by the prevention or proactive management of hypoglycaemia in a T1D population. In PwT2D, CGM may also have an important role in improving PROMs, particularly in those treated with insulin therapy. CSII is associated with improved PROMs in PwT1D, with limited evidence in a T2D population. HCL has been shown to have mixed outcomes across multiple studies, which may be because of differences between the HCL systems. Several studies of HCL recognize increased treatment satisfaction, improved QoL, and decreased diabetes‐related distress, but it is unclear whether this is because of a ‘class‐effect’ or individual systems.

While both CGM and HCL are becoming standard of care in T1D, the role of diabetes technology, specifically CSII and HCL, in T2D is less certain. Given the current range and future pipeline of T2D therapies, it may be that insulin use lessens over time. PwT2D may not prefer technology to administer insulin (with the associated risk of hypoglycaemia) over alternative pharmacotherapies, which have additional benefits for cardiovascular risk, weight loss or other co‐morbidities.^84^, ^85^ Looking to the future, understanding the impact of closed‐loop technology on the lived experience of people with T2D, particularly in comparison with pharmacological treatments, will be paramount.^86^, ^87^ Further insights into the impact of HCL and fully closed‐loop in PwT2D will be welcomed.

In conclusion, PROMs provide valuable insight into the impact of diabetes technologies on people living with diabetes. However, the range of PROMs used is broad and heterogeneous, making direct comparisons between technologies and, over time, difficult. Going forward, consensus and unity on the key PROMs to be reported in future studies would help improve consistency and ease of interpretation.

## AUTHOR CONTRIBUTIONS

ALL, TSJC and EGW edited, reviewed and critically revised the manuscript. All authors have approved the final version of the manuscript.

## FUNDING INFORMATION

ALL and TSJC received honoarium from Diabetes, Obesity and Metabolism for this article.

## CONFLICT OF INTEREST STATEMENT

ALL has received speaker fees and/or support to attend conferences from Dexcom and Novo Nordisk and research support from the Association of British Clinical Diabetologists. TSJC has received speaker fees and/or support to attend conferences from Eli Lilly, Novo Nordisk, Sanofi, Abbott Diabetes Care, Dexcom and Insulet. EGW has received personal fees from Abbott, AstraZeneca, Dexcom, Eli Lilly, Embecta, Glooko, Insulet, Medtronic, Novo Nordisk, Roche, Sanofi, Sinocare and Ypsomed, research support from the Association of British Clinical Diabetologists, Abbott, Diabetes UK, Embecta, Insulet, Novo Nordisk and Sanofi, medical writing support from Abbott, Eli Lilly and Embecta, and participated in consultancy/advisory board for Abbott, Dexcom, Eli Lilly, Embecta, Insulet, Medtronic, Novo Nordisk, Roche and Sanofi.

### PEER REVIEW

The peer review history for this article is available at https://www.webofscience.com/api/gateway/wos/peer‐review/10.1111/dom.15858.

## Data Availability

Data sharing is not applicable to this article as no new data were created or analyzed.
